# Supplementary material for: Papain Affects the Percentage and Morphology of Microglia in Hippocampal Neuron–Glial Cultures
Source: Brain Sci. 2025 Apr 24;15(5):442. doi: 10.3390/brainsci15050442 (PMC12109584; doi:10.3390/brainsci15050442)
Supplement: Supplementary file 1 [file brainsci-15-00442-s001.zip › brainsci-3501341-supplementary.pdf]

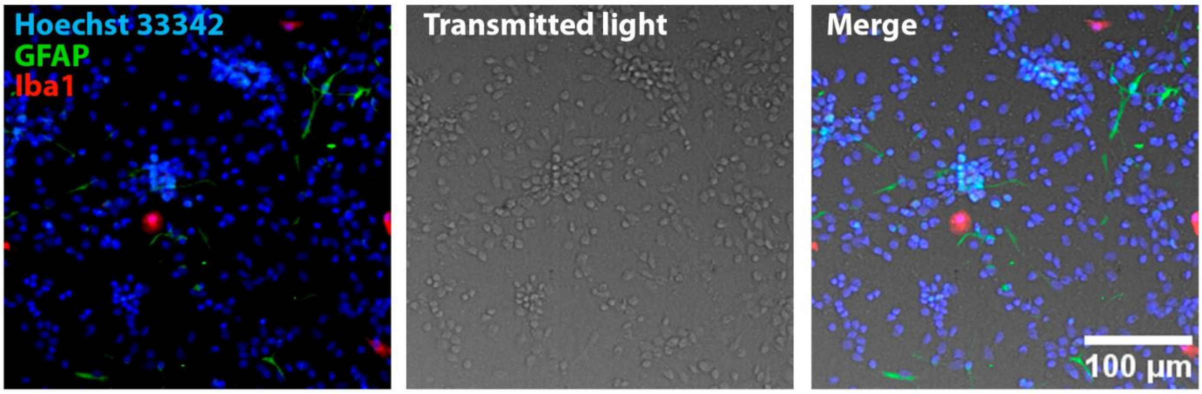

Figure S1. Staining of 2 DIV rat hippocampal TRY-cultures with antibodies against GFAP and Iba1.

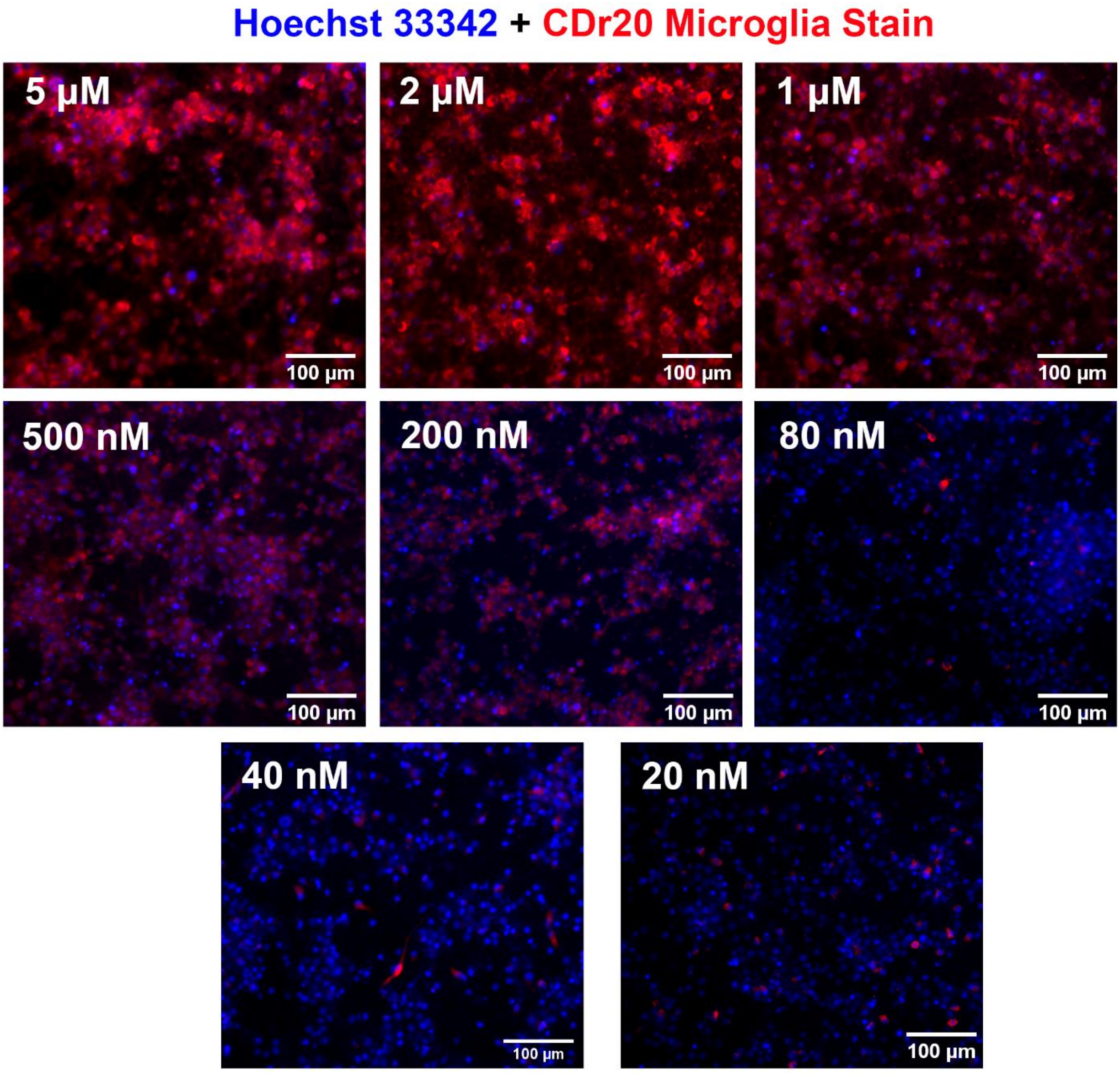

Figure S2. Staining of 7 DIV TRY-cultures with different concentrations of Lumi Cell CDr20 microglia stain. The nuclei were stained with Hoechst 33342 (5 µg/mL).

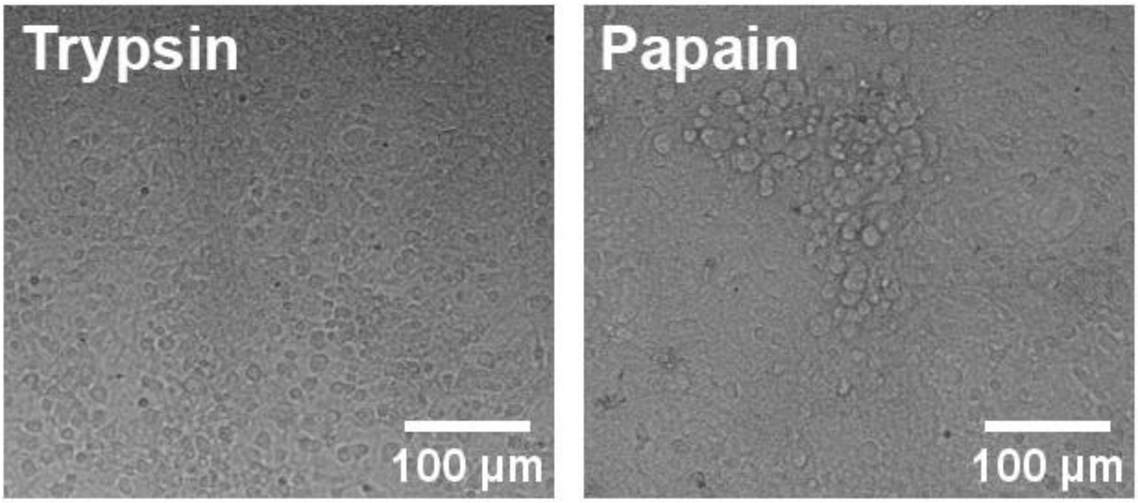

Figure S3. Transmitted light images of 7 DIV TRY- and PAP-cultures grown in the presence of TGFβ+MCSF+cholesterol.

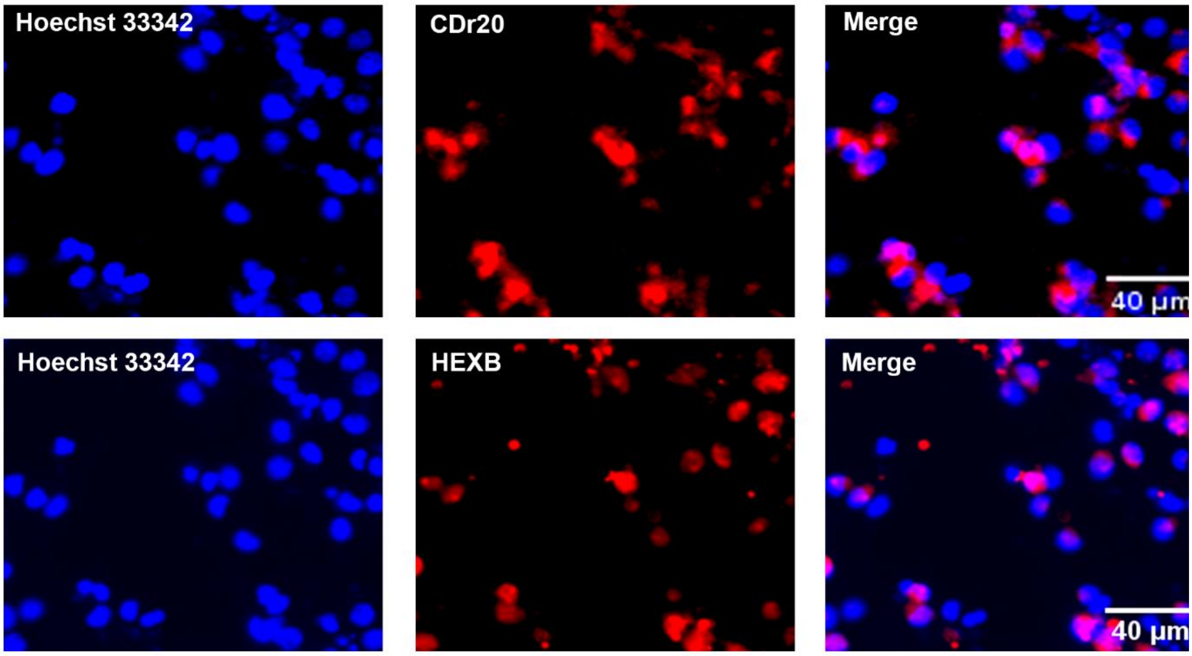

Figure S4. Images demonstrating the staining of 7 DIV PAP-culture with CDr20 and staining of the same culture area with antibodies to HEXB (1:300). The alignment of images before and after fixation was performed using a marker grid applied to the bottom of the glass with cultures, as described previously (10.1002/glia.23763).
